# Supplementary material for: Deep learning to estimate lithium-ion battery state of health without additional degradation experiments
Source: Nat Commun. 2023 May 13;14:2760. doi: 10.1038/s41467-023-38458-w (PMC10183024; doi:10.1038/s41467-023-38458-w)
Supplement: Supplementary file 1 — Supplementary Information [file 41467_2023_38458_MOESM1_ESM.pdf]

Supplementary Information

Deep learning to estimate lithium-ion battery state of health without additional degradation experiments

Jiahuan Lu[1], Rui Xiong[1], Jinpeng Tian[1], Chenxu Wang[1], Fengchun Sun[1]

[1]Department of Vehicle Engineering, School of Mechanical Engineering, Beijing Institute of Technology, Beijing 100081, China.

***Corresponding Authors*:** rxiong@bit.edu.cn (R. Xiong), tianjinpeng@bit.edu.cn (J. Tian)

Table S1 Degradation conditions of the selected lithium-ion batteries in this work.

Dataset	Cathode chemistry label	Nominal capacity (Ah)	Cell information	Test conditions
#1	LCO	1.1	3 cells termed CS2-35, CS2-36, CS2-37[1,2]	❖ Charged at a constant current rate of 0.5C until the voltage reached 4.2V and then 4.2V was sustained until the charging current dropped to below 0.05A.<br>❖ Discharged at a constant current rate of 1C.<br>❖ Ambient temperature is not mentioned.
#2	NMC	1.85	48 commercial UR18650E cylindrical cells[3]	❖ Charged at a constant current rate of 1C until the voltage reached 4.1V and then 4.1V was sustained until the charging current dropped to below 0.04A.<br>❖ Discharged at a constant current rate of 1C.<br>❖ 25°C.
#3	NCA	3.03	3 commercial NCR18650BD cylindrical cells	❖ Charged at a constant current rate of 0.3C until the voltage reached 4.2V and then 4.2V was sustained until the charging current dropped to below 0.03A.<br>❖ Discharged at a constant current rate of 2C.<br>❖ 20°C.
#4	LCO/NCO	0.74	8 KOKAM SLPB533459H 4 pouch cells[4]	❖ Charged at a constant current rate of 1C until the voltage reached 4.2V.<br>❖ Discharged at a constant current rate of 1C.<br>❖ 40°C.
#5	LFP	27	3 commercial IFP20100140A cells	❖ Charged at a constant current rate of 1C until the voltage reached 3.65V and then 3.65V was sustained until the charging current dropped to below 1.35A.<br>❖ Discharged at a constant current rate of 1C.<br>1. 45°C.

Supplementary Note 1. Estimation of additional experimental time for generating target labels

By neglecting the rest time during the battery degradation tests, we can use the current profile and capacity trajectories to estimate the minimum additional experimental time $\tau_{\min}$:

$$\tau_{\min} = \sum_{i=1}^{N_{\max}} \left(\frac{Q_{\mathrm{c},i}}{I_{\mathrm{c},i}} + \frac{Q_{\mathrm{d},i}}{I_{\mathrm{d},i}} \right)$$

where $N_{\max}$ is the total number of battery charge and discharge cycles. $Q_{\mathrm{c},i}$ and $Q_{\mathrm{d},i}$ are the charge capacity and discharge capacities of the i-th cycle, respectively. $I_{\mathrm{c},i}$ and $I_{\mathrm{d},i}$ are the charge current profile and discharge current profile of the i-th cycle, respectively. Assuming that there are enough channels for testing batteries, we can employ the largest $\tau_{\min}$ to represent the time required to generate the target domain labels.

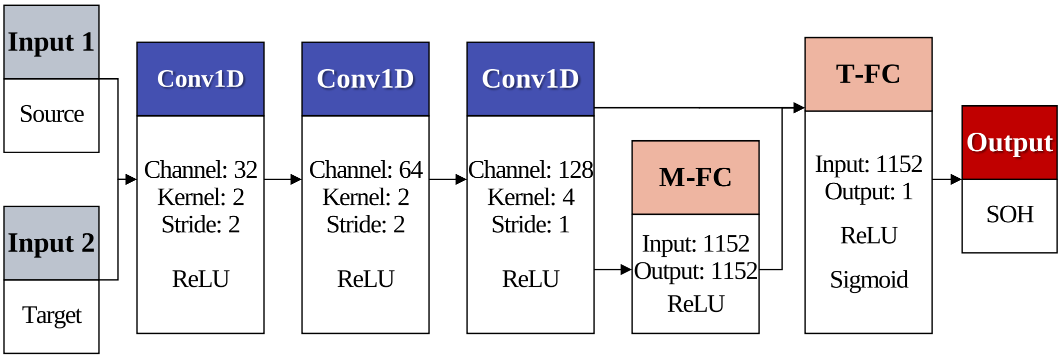

Fig. S1 Hyper-parameter settings for the DNN in the proposed framework

Table S2 Detailed scheme of cross-validation.

Target domain / Source domain	#1	#2	#3	#4	#5
#1		Combination 1:<br>Case 1-1: ~95%<br>Case 1-2: ~90%<br>Case 1-3: ~85%<br>Case 1-4: ~80%	Combination 2:<br>Case 2-1: ~95%<br>Case 2-2: ~90%<br>Case 2-3: ~85%<br>Case 2-4: ~80%	Combination 3:<br>Case 3-1: ~95%<br>Case 3-2: ~90%<br>Case 3-3: ~85%<br>Case 3-4: ~80%	Combination 4:<br>Case 4-1: ~95%<br>Case 4-2: ~90%<br>Case 4-3: ~85%<br>Case 4-4: ~80%
#2	Combination 5:<br>Case 5-1: ~95%<br>Case 5-2: ~90%<br>Case 5-3: ~85%<br>Case 5-4: ~80%		Combination 6:<br>Case 6-1: ~95%<br>Case 6-2: ~90%<br>Case 6-3: ~85%<br>Case 6-4: ~80%	Combination 7:<br>Case 7-1: ~95%<br>Case 7-2: ~90%<br>Case 7-3: ~85%<br>Case 7-4: ~80%	Combination 8:<br>Case 8-1: ~95%<br>Case 8-2: ~90%<br>Case 8-3: ~85%<br>Case 8-4: ~80%
#3	Combination 9:<br>Case 9-1: ~95%<br>Case 9-2: ~90%<br>Case 9-3: ~85%<br>Case 9-4: ~80%	Combination 10:<br>Case 10-1: ~95%<br>Case 10-2: ~90%<br>Case 10-3: ~85%<br>Case 10-4: ~80%		Combination 11:<br>Case 11-1: ~95%<br>Case 11-2: ~90%<br>Case 11-3: ~85%<br>Case 11-4: ~80%	Combination 12:<br>Case 12-1: ~95%<br>Case 12-2: ~90%<br>Case 12-3: ~85%<br>Case 12-4: ~80%
#4	Combination 13:<br>Case 13-1: ~95%<br>Case 13-2: ~90%<br>Case 13-3: ~85%<br>Case 13-4: ~80%	Combination 14:<br>Case 14-1: ~95%<br>Case 14-2: ~90%<br>Case 14-3: ~85%<br>Case 14-4: ~80%	Combination 15:<br>Case 15-1: ~95%<br>Case 15-2: ~90%<br>Case 15-3: ~85%<br>Case 15-4: ~80%		Combination 16:<br>Case 16-1: ~95%<br>Case 16-2: ~90%<br>Case 16-3: ~85%<br>Case 16-4: ~80%
#5	Combination 17:<br>Case 17-1: ~95%<br>Case 17-2: ~90%<br>Case 17-3: ~85%<br>Case 17-4: ~80%	Combination 18:<br>Case 18-1: ~95%<br>Case 18-2: ~90%<br>Case 18-3: ~85%<br>Case 18-4: ~80%	Combination 19:<br>Case 19-1: ~95%<br>Case 19-2: ~90%<br>Case 19-3: ~85%<br>Case 19-4: ~80%	Combination 20:<br>Case 20-1: ~95%<br>Case 20-2: ~90%<br>Case 20-3: ~85%<br>Case 20-4: ~80%	

Supplementary Note 2. Impact of different voltage windows

The impact of the voltage window length is investigated by varying the window length from 300 to 800 mV with steps of 50 mV. Each case contains validation over 71,588 cycles collected from 65 commercial LIB cells produced by five different manufacturers. Sampling for each window length terminates at 4 V for Cell #1~4 and 3.6 V for Cell #5. The absolute error distribution of the SOH estimation in the cases is shown in Fig. S2. It can be noticed that the proposed framework achieves accurate estimation with an absolute error below 2.4% in most of the cases at the voltage window from 450 mV to 800 mV. The absolute error increases as the window length decrease due to less available input information. Even if we use the data at the voltage window as less than 350 mV, the proposed framework can give reliable estimates, with most of the absolute errors lying under 4%. One might need to determine an appropriate window length to balance estimation accuracy and sampling duration according to application scenarios.

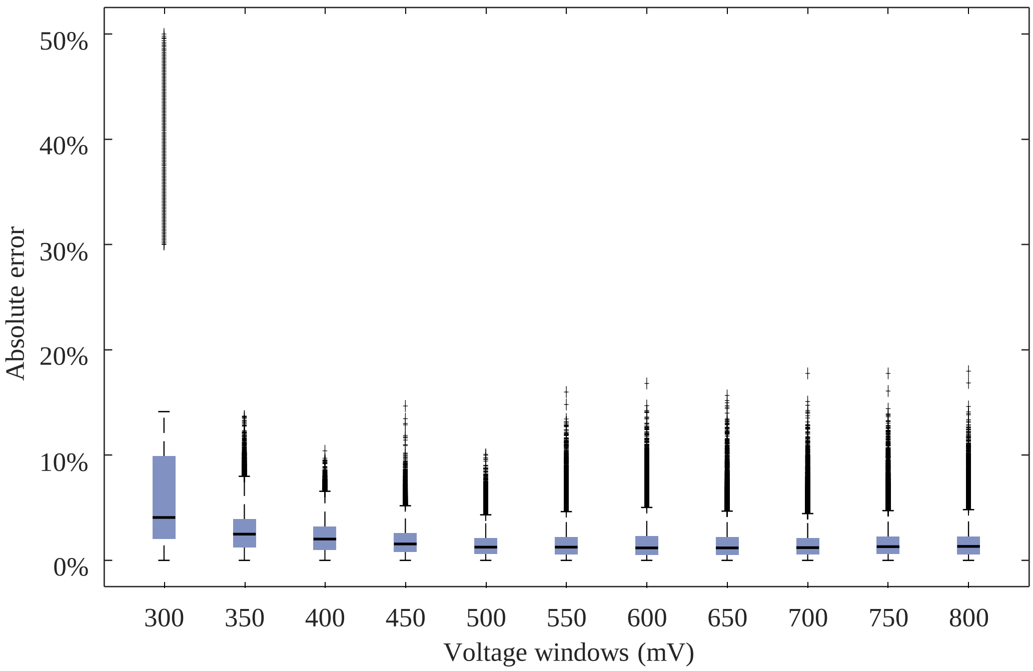

Fig. S2 Distribution of absolute errors for estimates using different voltage windows.

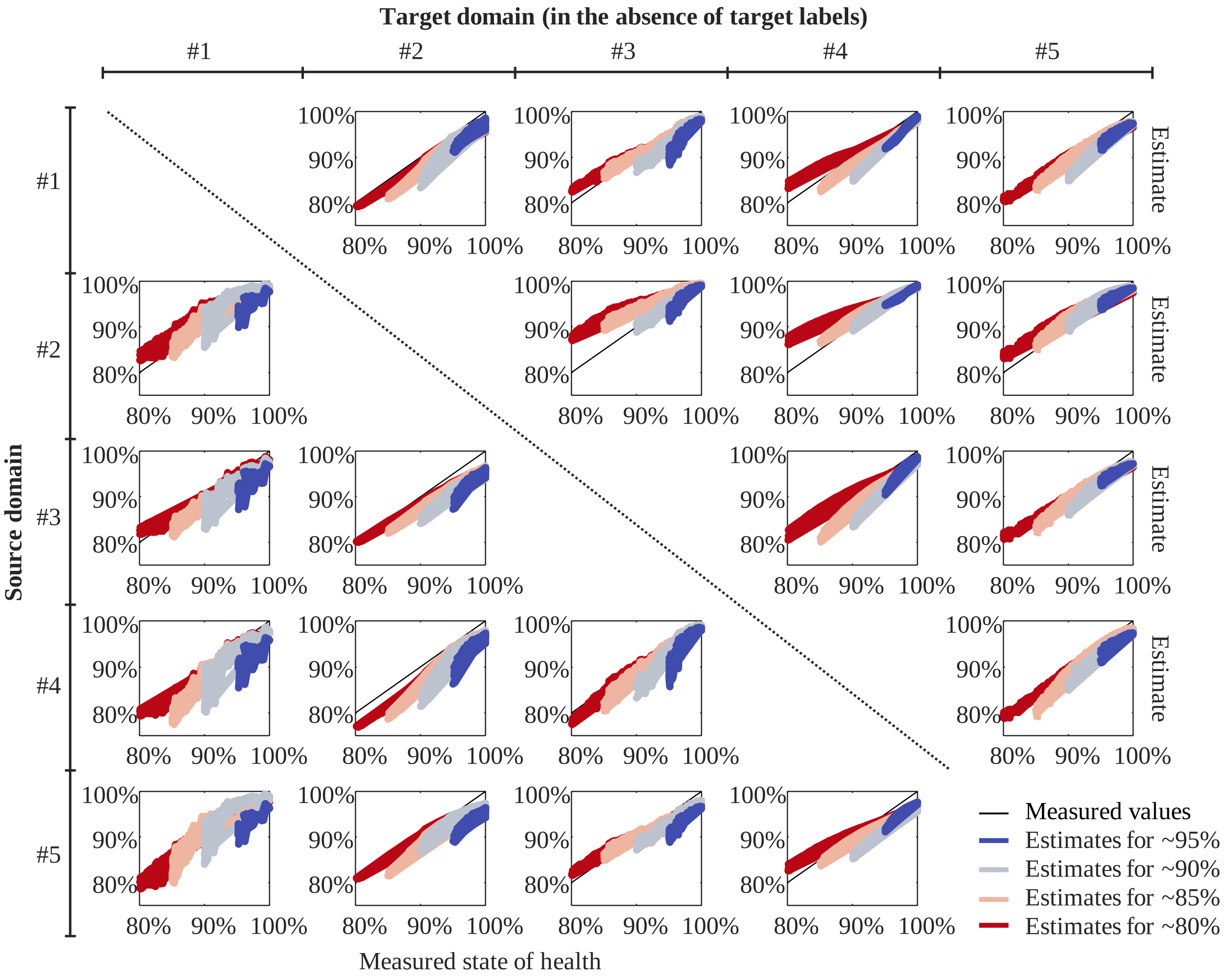

Fig. S3 Results of cross-manufacturer battery SOH estimation before the trim in the absence of target labels.

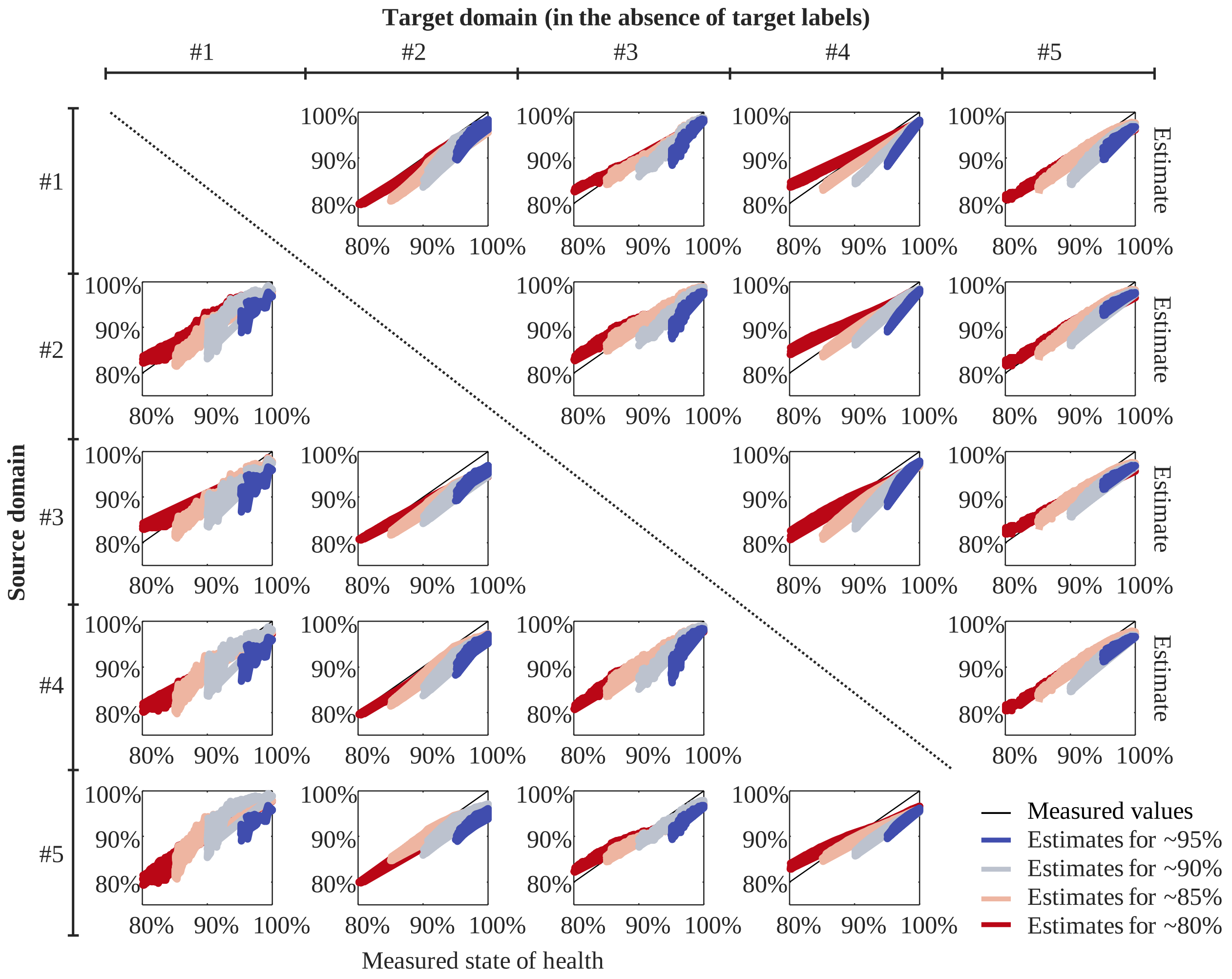

Fig. S4 Results of cross-manufacturer battery SOH estimation after the trim in the absence of target labels.

Table S3 Settings for the methods involved in the comparison.

Method	Key hyper-parameters
Gaussian process regression (GPR)[5]	Covariance function: Matérn kernel with parameter 5/2<br>Mean function: None
Random forest regression (RF)[6]	Number of decision trees: 500<br>Number of decision splits for each predictor: one-third of the number of all predictor variables
Support vector regression (SVR)[7]	Kernel function: radial basis kernel function<br>Number of training subsets for bagging strategy: 10<br>Number of folds to use in a cross-validated model: 5
Convolutional neural network (CNN)[8]	Numbers of CNN blocks (Conv(3,4,1) + Batch normalization + ReLU): 5[*]<br>Numbers of CNN blocks (Conv(3,8,1) + Batch normalization + ReLU): 4[*]<br>Numbers of CNN blocks (Conv(3,16,1) + Batch normalization + ReLU): 4[*]<br>Numbers of global average pooling: 1<br>Numbers of Dense(1) blocks: 1[*]<br>State balancing factor: 1<br>Maximum number of training epochs: 2000
Benchmark 1	A single DNN with a comparable number of hyperparameters to the proposed framework, which can be graphically described as:<br>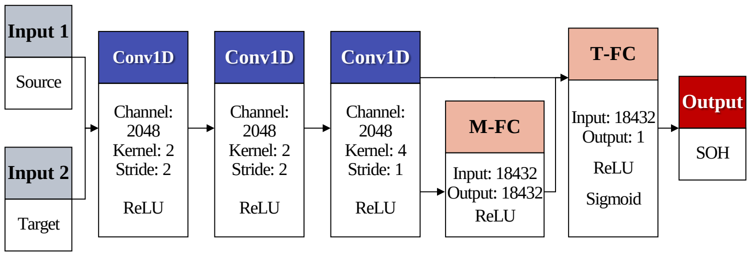<br>The rest of the settings are consistent with the proposed framework
Benchmark 2	A framework that equally weights the DNN swarm (the size is 300)<br>The weight κ_3 of the loss function is set to 0<br>The rest of the settings are consistent with the proposed framework
Benchmark 3	A single DNN of the framework<br>The weight κ_3 of the loss function is set to 0<br>The rest of the settings are consistent with the proposed framework

* Conv (x, y, z) denotes a 1D CNN layer that has a kernel size of x, the number of filters of y and a stride of z. ReLU denotes the ReLU activation. Dense (x) denotes a dense layer with x neurons.

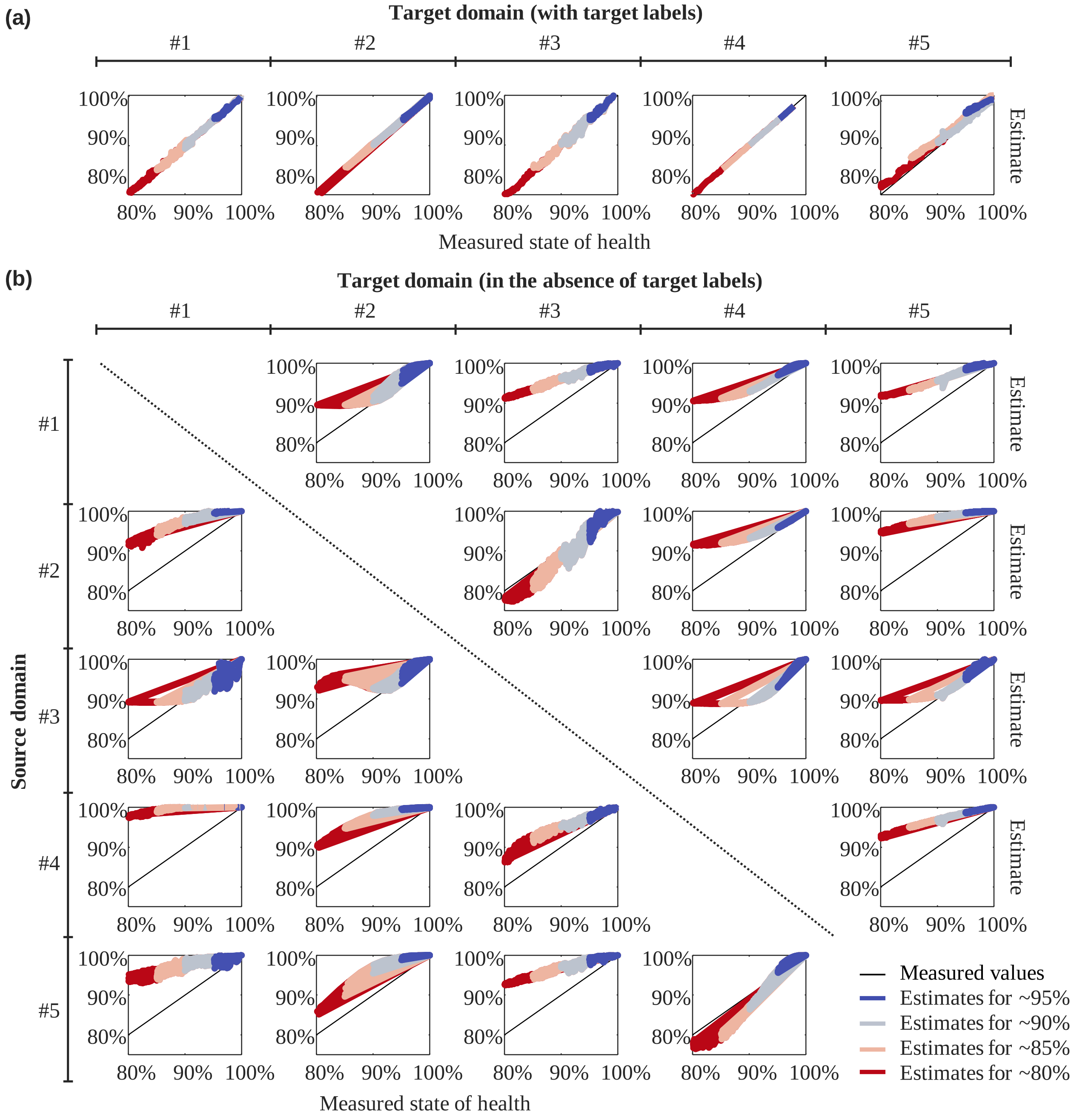

Fig. S5 Results of the Gaussian process regression (GPR).

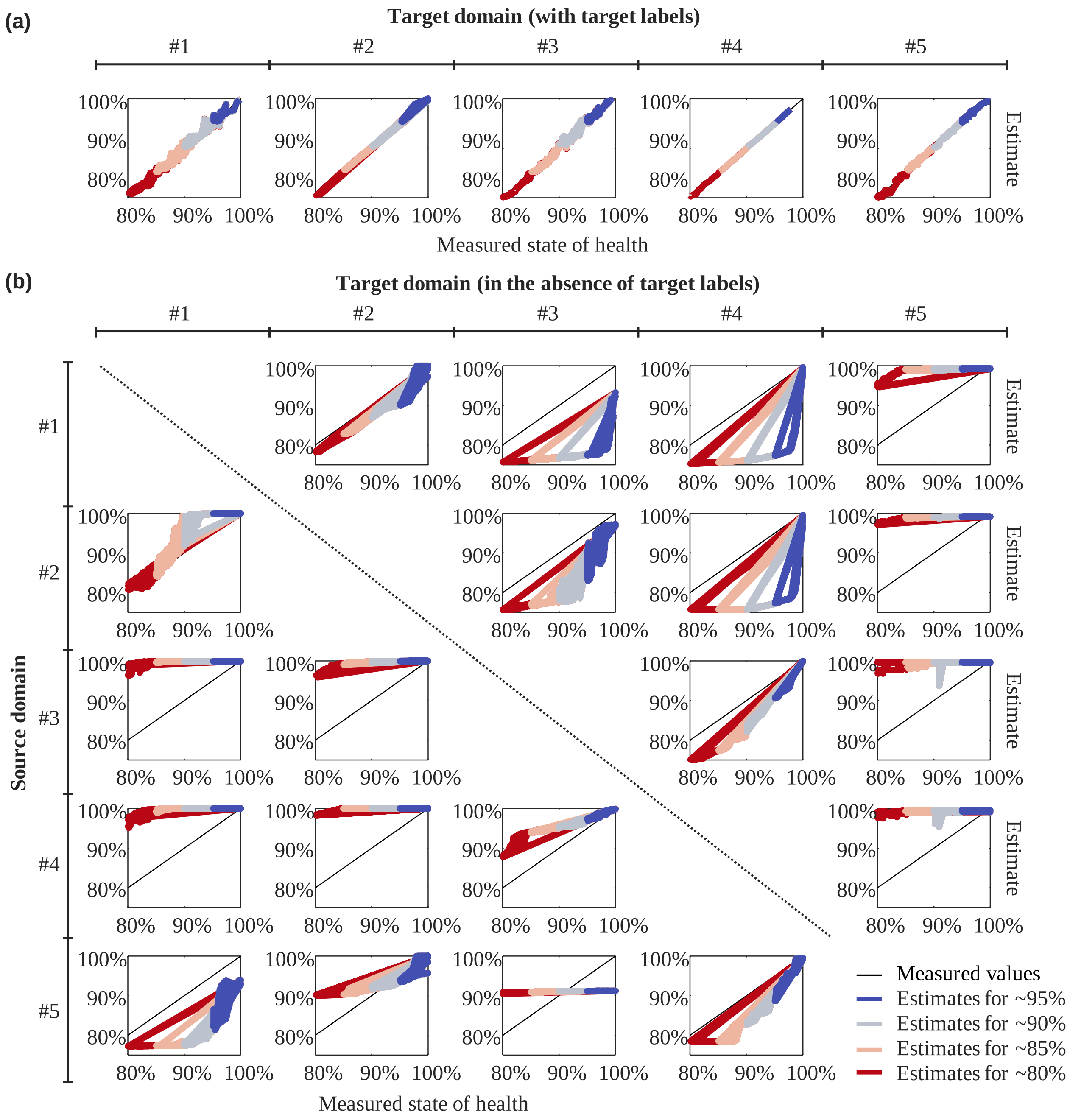

Fig. S6 Results of the random forest (RF).

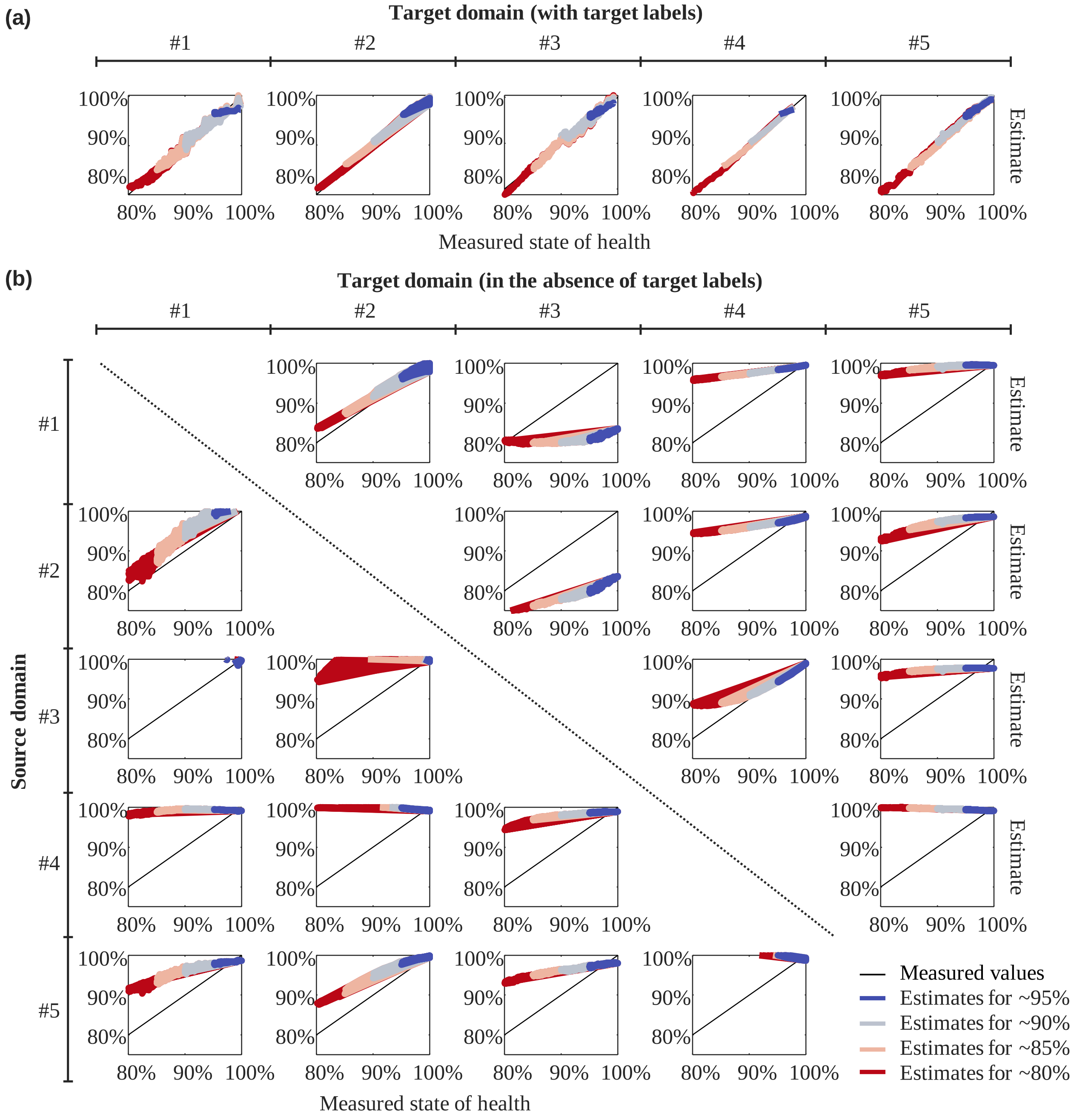

Fig. S7 Results of the support vector regression (SVR).

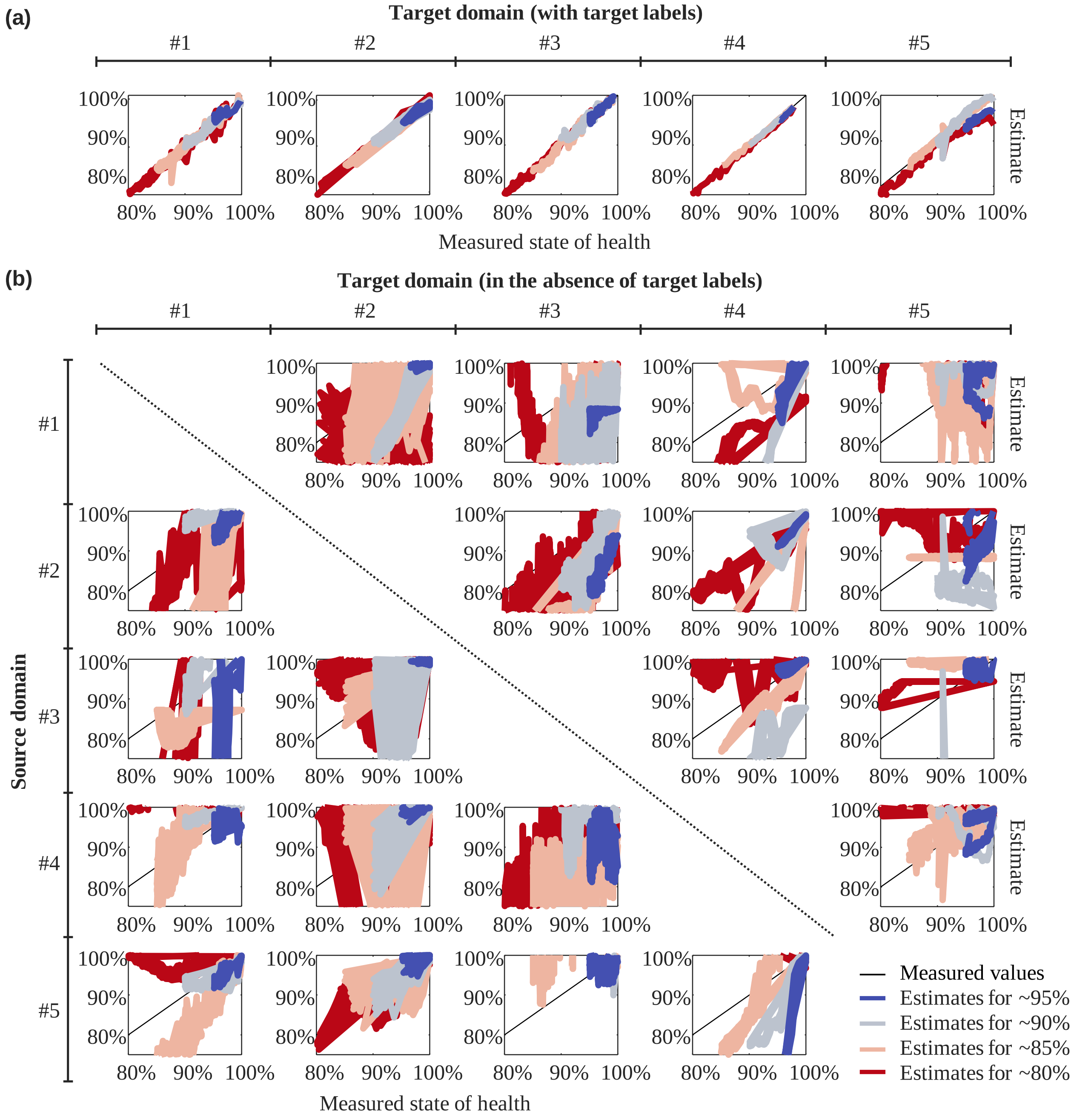

Fig. S8 Results of the convolutional neural network (CNN).

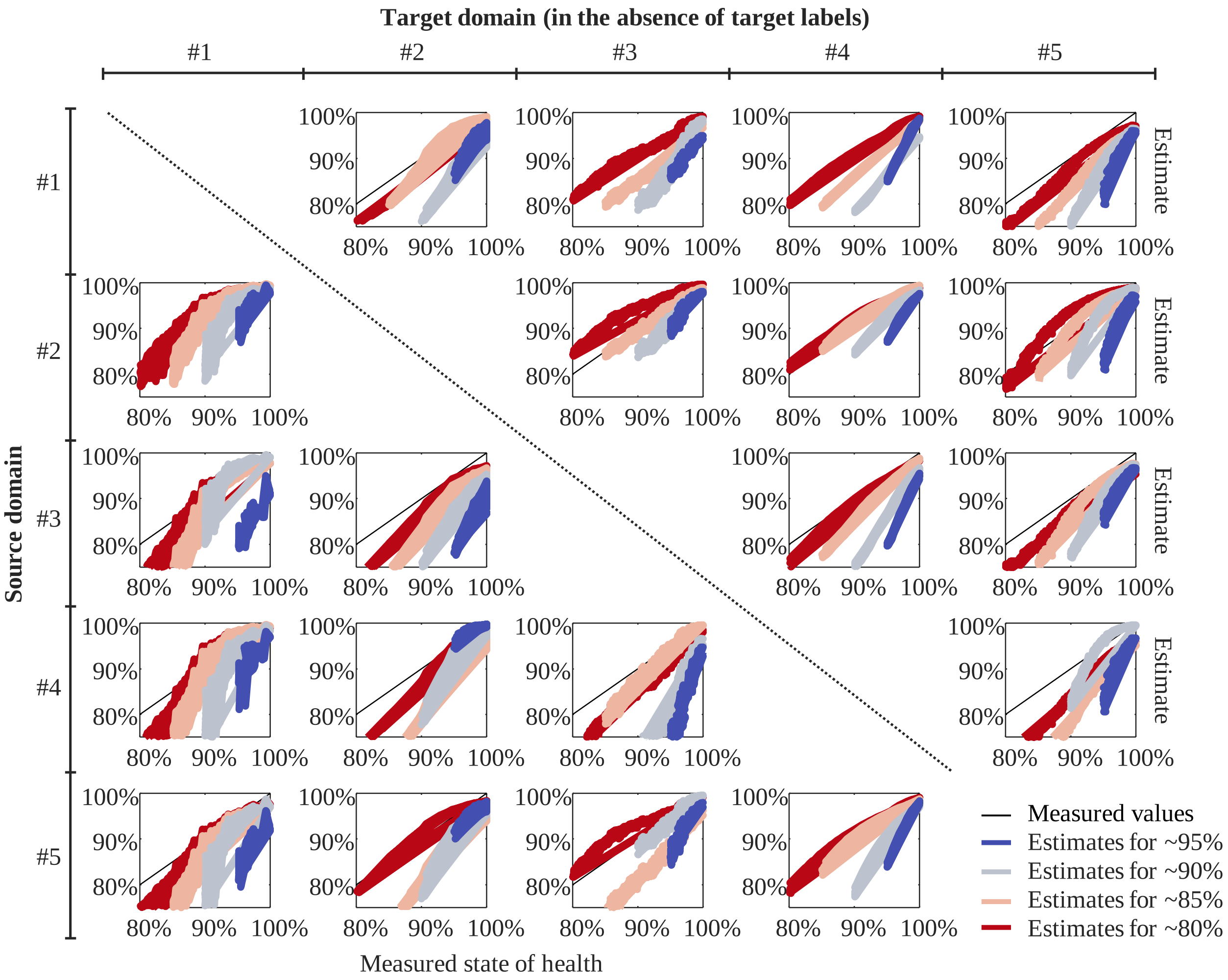

Fig. S9 Results of the Benchmark (BM) 1.

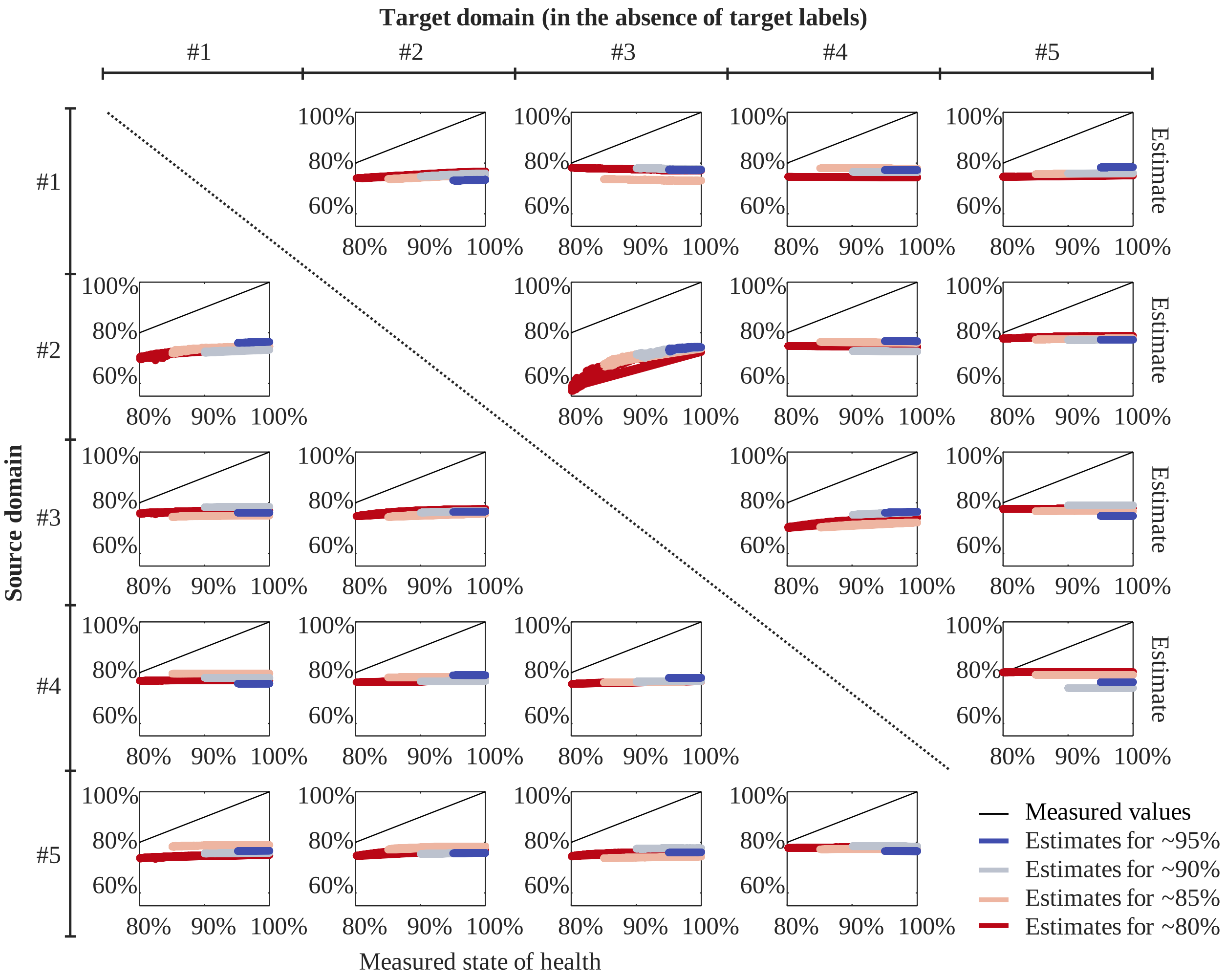

Fig. S10 Results of the BM 2.

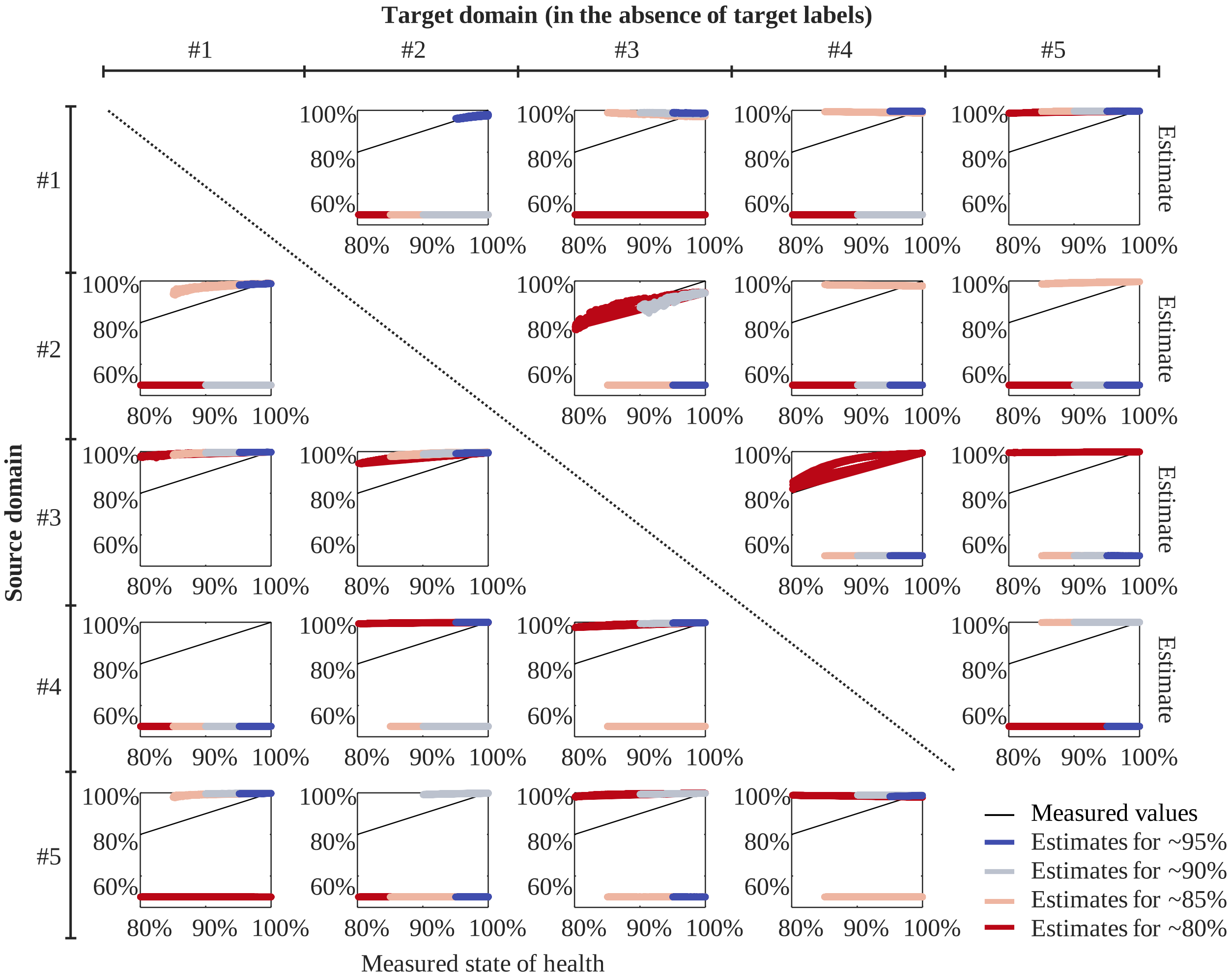

Fig. S11 Results of the BM 3.

Supplementary Note 3. Comparison of the computational cost

The computational costs (averaged over all cases) of the involved methods (i.e., GPR, RF, SVR, CNN, and the proposed framework) for training in the absence of target labels are listed in Table S4. We observe that the existing methods have advantages in computational cost due to their rough structures, but their accuracy in the absence of target labels is practically unacceptable as mentioned in "Comparison with existing methods". Instead, the proposed framework takes merely about 0.7 hours to train 300 DNNs, showing an acceptable computational cost. More importantly, our framework can avoid conducting 644-8473 hours of degradation experiments. As mentioned in "Estimation performance with various hyper-parameters", a swarm size of 50 is sufficient for accurate estimation. Thus, under the premise of guaranteeing accuracy, one might employ a small swarm size to further reduce the computational cost in practice.

Table S4 Computational cost of the involved methods in the absence of target labels.

Method	GPR	RF	SVR	CNN	Proposed (Swarm size: 300)
Computational cost (hours)	0.0022	0.0014	0.0008	0.0185	0.7098

Supplementary Note 4. Analysis of the pre-estimate distribution of DNN swarm

We find that, for a given batch of target domain samples, the estimation results of the DNNs in the swarm are diverse, but their distributions show regularity with their accuracy. To demonstrate this, we take a pair of instances in the ~85% cases (i.e., the cases of transfer from Dataset #1 to #5 and from Dataset #5 to #1) to study the pre-estimate distribution of DNN swarm (see Fig. S12). We observe that most DNNs make underestimates (represented by a right shift in Fig. S12). This is primarily affected by domain imbalance: the SOH distribution of the source domain (e.g., distributed from 100% to 75%) is generally more comprehensive than that of the target domain (e.g., distributed from 100% to 85% before reaching the end of life). Besides, some DNNs seem to be trained badly, making abnormally low estimates (concentrated at the far right of Fig. S12). Thus, selecting those DNNs with high estimates in the swarm is beneficial for accurate estimation. This motivates us to formulate a criterion for selecting well-performing DNNs from the swarm by measuring the statistical characteristics of each DNN's pre-estimates.

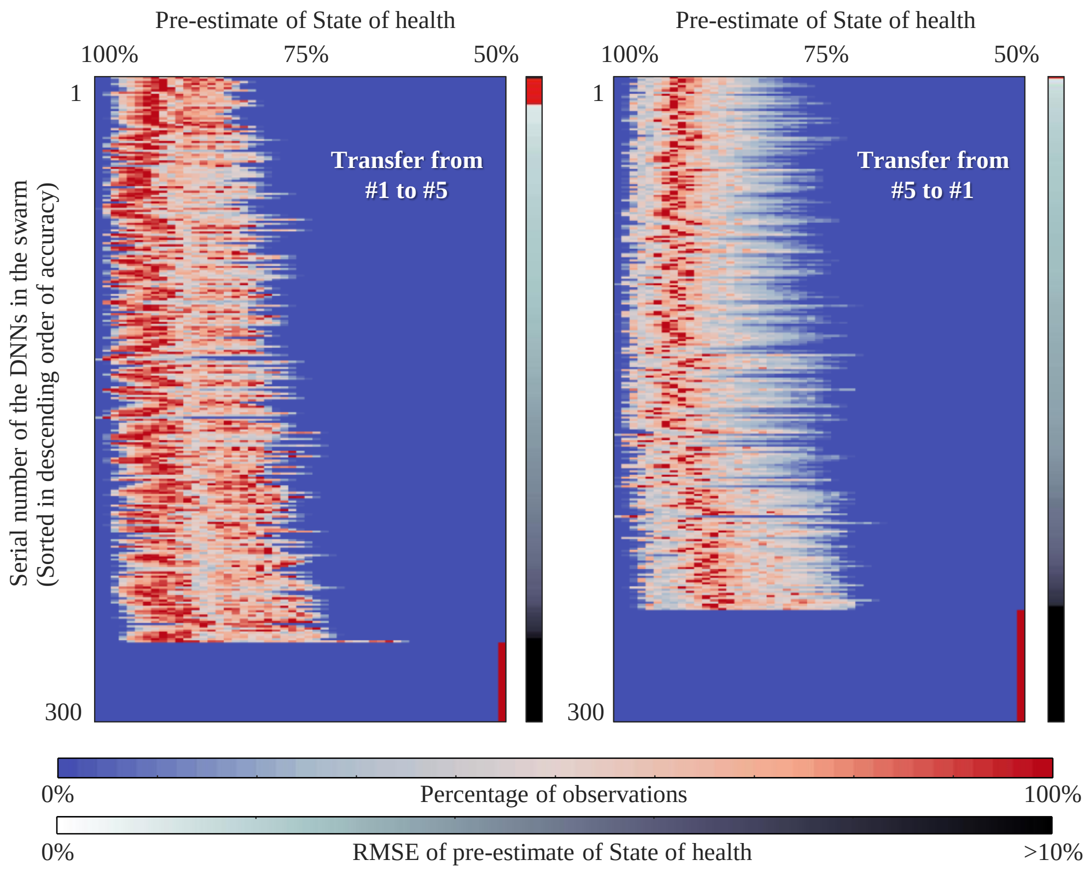

Fig. S12 Pre-estimate distribution of the DNN swarm in the proposed framework in the ~85% case (i.e., cases of transfer from Dataset #1 to #5 and from Dataset #5 to #1)

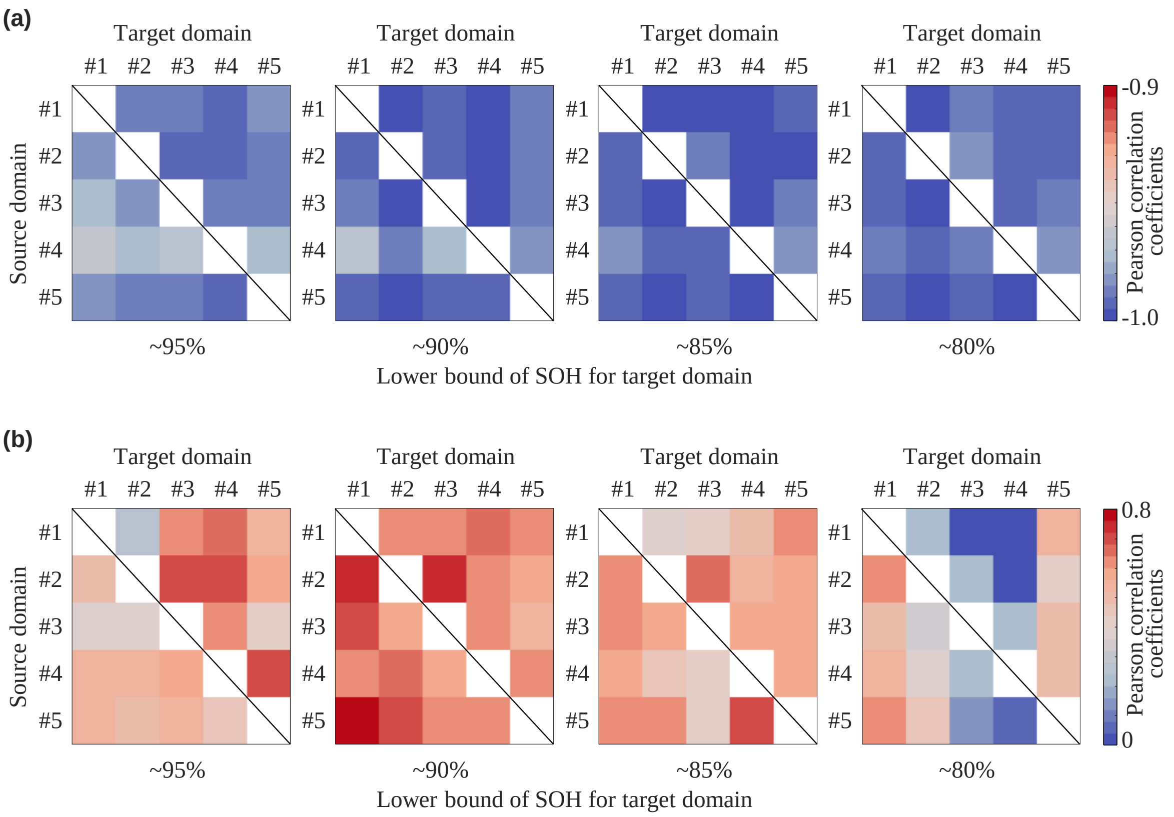

Fig. S13 Pearson correlation coefficients over 80 cases. (a) Correlation coefficients between the RMSE and the mean of the DNN pre-estimates. (b) Correlation coefficients between the RMSE and the standard deviation of the DNN pre-estimates.

Supplementary Reference

1. He, W., Williard, N., Osterman, M. & Pecht, M. Prognostics of lithium-ion batteries based on Dempster-Shafer theory and the Bayesian Monte Carlo method. *J Power Sources* **196**, 10314–10321 (2011).
2. Xing, Y., Ma, E. W. M., Tsui, K. L. & Pecht, M. An ensemble model for predicting the remaining useful performance of lithium-ion batteries. *Microelectron Reliab* **53**, 811–820 (2013).
3. Li, W. *et al.* One-shot battery degradation trajectory prediction with deep learning. *J Power Sources* **506**, (2021).
4. Birkl, C. Oxford battery degradation dataset 1. *University of Oxford* (2017).
5. Richardson, R. R., Birkl, C. R., Osborne, M. A. & Howey, D. A. Gaussian Process Regression for in Situ Capacity Estimation of Lithium-Ion Batteries. *IEEE Trans Industr Inform* **15**, 127–138 (2019).
6. Li, Y. *et al.* Random forest regression for online capacity estimation of lithium-ion batteries. *Appl Energy* **232**, 197–210 (2018).
7. Guo, Y., Huang, K., Yu, X. & Wang, Y. State-of-health estimation for lithium-ion batteries based on historical dependency of charging data and ensemble SVR. *Electrochim Acta* **428**, 140940 (2022).
8. Tian, J., Xiong, R., Shen, W., Lu, J. & Sun, F. Flexible battery state of health and state of charge estimation using partial charging data and deep learning. *Energy Storage Mater* **51**, 372–381 (2022).
